# Comparison of Gut Microbiomes Between Neonates Born by Cesarean Section and Vaginal Delivery: Prospective Observational Study

**DOI:** 10.1155/bmri/8302361

**Published:** 2024-11-28

**Authors:** Nichapat Pahirah, Amarin Narkwichean, Malai Taweechotipatr, Sivaporn Wannaiampikul, Chinpanee Duang-Udom, Wipada Laosooksathit

**Affiliations:** ^1^Department of Obstetrics and Gynecology, Faculty of Medicine, Srinakharinwirot University, Nakhon Nayok, Thailand; ^2^Department of Microbiology, Faculty of Medicine, Srinakharinwirot University, Bangkok, Thailand; ^3^Department of Biochemistry, Faculty of Medicine, Srinakharinwirot University, Bangkok, Thailand; ^4^Faculty of Medicine, Srinakharinwirot University, Nakhon Nayok, Thailand

**Keywords:** 16S rRNA, cesarean section, gut microbiome, neonates, vaginal delivery

## Abstract

**Background:** Balanced diversity and abundance of gut microbiome play important roles in human health, including neonatal health. Though not established, there is evidence that the delivery route could alter the diversity of neonatal gut microbiomes.

**Objective:** The objective of the study was to investigate the differences in the gut microbiomes of neonates delivered via cesarean section compared to those born by vaginal delivery and to identify the predominant microbial taxa present in each group.

**Study Design:** A prospective observational study of 281 healthy neonates born between February 2021 and April 2023 at Her Royal Highness Maha Chakri Sirindhorn Medical Center, Srinakharinwirot University, Thailand, was performed. The study population was divided into two groups: 139 neonates born via vaginal delivery and 141 neonates born via cesarean section. The microbiota composition of each neonate's fecal sample was identified by using 16S ribosomal ribonucleic acid metagenomic sequencing.

**Results:** Neonates delivered vaginally exhibited a gut microbiome with higher abundance and diversity than those delivered by cesarean delivery. *Bifidobacterium* was the dominant genus in both groups. *Bifidobacterium breve* was the dominant species and was significantly higher in cesarean-delivered neonates compared to those delivered vaginally (24.0% and 9.2%, respectively) (*p* < 0.001). However, the taxonomy of only 89 (64.0%) and 44 (31.43%) fecal samples could be identified from the vaginal and cesarean delivery groups, respectively.

**Conclusion:** Route of delivery is associated with neonatal gut microbiome abundance and diversity. Neonates delivered via vaginal delivery exhibited higher diversity but lower abundance of the dominant species in the gut microbiome.

**Trial Registration:** Thai Clinical Trials Registry identifier: TCTR20221024003

## 1. Introduction

Human gut microbiomes are the collection of bacteria, viruses, fungi, protozoa, and eukaryotes colonizing the human gastrointestinal tract. These microbiomes have high functional capacity and benefit the host in several ways. The diversity of gut microbiomes is shaped by the host and many environmental factors [1, 2]. Genetics, mode of delivery, diet, population, and location can diversify these gut microbes [1–4]. Loss of gut microbiome diversity and balance can cause diabetes, allergies, autoimmune diseases, and obesity [2, 5].

The changes in gut microbiome diversity during pregnancy increase some risks of pregnancy complications, such as gestational diabetes, preeclampsia, maternal infection, growth restriction, and intrauterine demise [4, 6]. Common bacteria in neonatal gut microbiomes are Actinobacteria (*Bifidobacterium*) [7], Firmicutes [7, 8], and *Lactobacillus* [9]. Studies show a relationship between maternal and neonatal gut microbiome diversity [4, 6]. The gut microbes can pass from mothers to babies via many routes, such as via the vagina, the gastrointestinal tract, the skin, and breast milk, and subsequently colonize the infant's gut immediately after birth [4, 8]. The infant's immune system is an important system that can be affected by this gut microbiota diversity and potentially affect long-term outcomes [10]. Because the vagina is one of the routes by which mothers pass microbiotas on to their babies, the question of whether the route of delivery can affect the infant's microbiome can be posed.

The cesarean section rate has been increasing over the last few decades. Data from the World Health Organization (WHO) in 2021 showed the cesarean rate was 21%, compared with 7% in 1990 [11]. Infants born via cesarean section have different exposures to substances from those born vaginally [5]. There has been conflict regarding whether the mode of delivery can alter a newborn's gut microbiome diversity.

There are many methods widely used to identify gut microbiomes, one of which is metagenomic sequencing. Using a 16S ribosomal ribonucleic acid (rRNA) gene sequencing technique, uncultured microbiomes can be identified. It can provide information on the complete genome in less time and is more accurate than the classic method. This method is widely used today [12, 13].

Attempts to study neonatal outcomes of cesarean delivery related to gut microbiomes in early life have been made and are increasing in many countries. Yet, there have been few studies carried out in Thailand. Understanding the differences in neonatal gut microbiomes between those delivered via vaginal delivery and those delivered by cesarean section in the Thai population, including the factors that can affect the diversity of gut microbiomes, may help researchers identify risk factors, preventive methods, or interventions to help promote the long-term outcomes of those delivered via cesarean section.

## 2. Methods

A prospective study was aimed at investigating the differences in gut microbiomes between neonates born via vaginal and cesarean delivery. Term neonates born at the Srinakharinwirot University Hospital between February 2022 and April 2023 were included. The study was approved by the Institutional Review Board (SWUEC-M/029/2564E).

**Figure 1:**
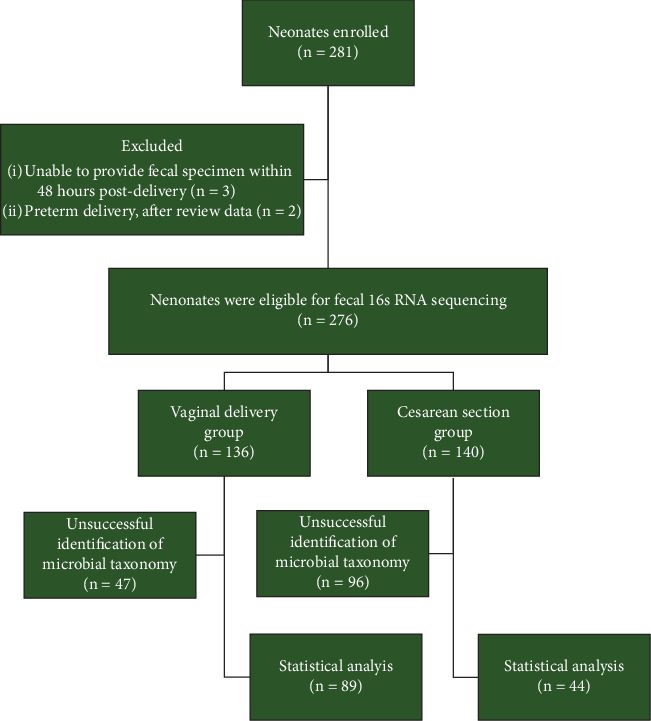
Flow chart of the study enrollment process.

All healthy-term neonates delivered at the institution were included. We excluded neonates born to mothers with prior infections, those whose mothers received antibiotics for cesarean delivery (except for preoperative prophylaxis), those unable to provide a fecal specimen within 48 h postdelivery, and those with neonatal complications or previous antibiotic use. The flow chart of participants enrolled in the study is presented in Figure 1. Written consent was obtained from the mothers, and each patient's demographic data, route of delivery, neonatal outcomes, and timing of fecal collection were collected.

**Figure 2:**
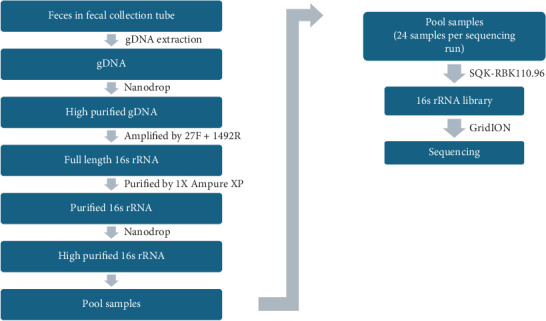
Steps of the process performed.

The study processes after data collection were fecal collection, genomic DNA (gDNA) extraction, purification, 16S rRNA amplification, 16S rRNA library preparation, and sequencing (Figure 2).

### 2.1. Fecal Collection

All fecal samples were collected within 48 h using Zymo DNA/RNA Shield fecal collection tubes (Zymo Research, United States). At least 1 g or 1 mL of neonates' feces was collected, mixed, and then stored at 2°C–25°C before being transferred into a −80°C freezer for storage. The date and time of fecal sample collection were recorded to determine the neonatal age at the time of sample collection.

### 2.2. gDNA Extraction

gDNA was extracted from approximately 100 mg of each fecal sample using the ZymoBIOMICS DNA Miniprep Kit (Zymo Research, United States) according to the manufacturer's protocol. A NanoDrop spectrophotometer was used to quantify and assess the purification of the gDNA. The expected real-time quality score had to be ≥ 7. However, the results obtained using the given protocol showed low DNA concentrations and inadequate purification for amplification. Therefore, we adjusted the protocol to achieve a better yield and more highly purified gDNA (see the Supporting Information section (available here)).

### 2.3. 16S rRNA Amplification

The full length of 16S rRNA was amplified using 50 ng of gDNA with Q5 High-Fidelity 2X Master Mix (New England Biolabs, MA, United States) and the universal primers 27F (5⁣′-AGRGTTYGATYMTGGCTCAG-3⁣′) and 1492R (5⁣′-CGGYTACCTTGTTACGACTT-3⁣′) (Lane, 1991). The polymerase chain reaction (PCR) conditions were 98°C for 30 s, 35 cycles at 98°C for 10 s, 55°C for 30 s, 72°C for 1 min, and 72°C for 10 min. The PCR products were purified and concentrated using the FavorPrep GEL/PCR Purification Mini Kit (Medibena, Austria). The purified PCR products were quantified and checked for purity by the NanoDrop machine (Thermo Fisher Scientific, United States). The concentration of purified PCR products was adjusted to 200 ng in 10 *μ*L nuclease-free water.

### 2.4. 16S rRNA Library Preparation and Sequencing

The 16S rRNA libraries were prepared using a Rapid Barcoding Kit 24V14 (SQK-RBK114.24) (Oxford Nanopore Technologies, United Kingdom) according to the manufacturer's protocol. The 24 barcodes were pooled in equal concentrations. The sequencing was performed using a MinION Flow Cell (R10.4.1) with a GridION sequencer (Oxford Nanopore Technologies, United Kingdom).

### 2.5. 16S rRNA Bioinformatic Analysis

**Figure 3:**
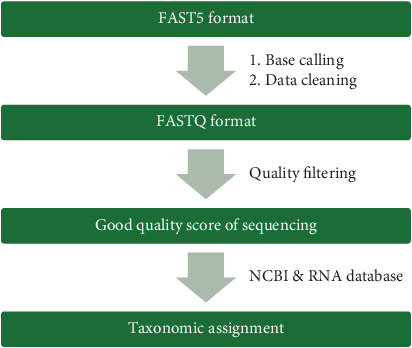
Data analysis of next-generation sequencing workflow.

The 16S rRNA analysis workflow is shown in Figure 3. The results from the next-generation sequencing were in the FAST5 format. The FastQ files were generated from the results in the FAST5 format by using the Guppy program in MinKNOW (Oxford Nanopore Technologies, United Kingdom), in which real-time quality scores of more than 7 were filtered and demultiplexed. The FastQ passing quality was aligned to reference sequences in the NCBI database by using the FASTQ 16S workflow (Version 2022.01.07), and the inclusion criteria were a minimum BLAST *e*-value of 0.01, minimum coverage of more than 30%, and a minimum identity of more than 77%. The data output files were generated as CSV files. Then, the percentage prevalence of each species and genus was calculated, and species and genus were filtered at a level of 0.5% for investigation of relative abundance.

Comparative taxonomy was performed to investigate the relative abundance of species between neonates delivered vaginally and those delivered by cesarean delivery.

### 2.6. Statistical Analysis

The sample size was calculated using the formula for comparing two independent means, based on the mean (standard deviation [SD]) prevalence of gut *Bifidobacterium* of infants delivered by vaginal and cesarean deliveries (36.6% [33.0%] vs. 48.6% [36.3%]) [14]. The sample size for each group was calculated to be at least 131. An additional 10 samples were added to each group to compensate for exclusions, resulting in a total of 282 samples being required.

The SPSS Version 29.0.2.0 (IBM Corp., New York) was used for statistical analysis. The baseline characteristics were analyzed using descriptive statistics, that is, mean ± SD, median with an interquartile range, and percentage, as appropriate. Beta diversity was used to demonstrate the difference in diversity between groups. The independent *T*-test with unequal variance statistic was used to compare the statistical differences in gut microbiome between neonates delivered by vaginal delivery and those delivered by cesarean delivery.

## 3. Results

A total of 281 neonates were enrolled in the study, consisting of 139 and 142 neonates in the vaginal delivery group (VG) and the cesarean section group (CS), respectively. Three cases from the VG were excluded due to the inability to provide a fecal sample within 48 h, and two cases from the CS were excluded due to preterm delivery after a review of data. The study flow is shown in Figure 1. The mean (SD) fecal collection times were 18.6 (12.1) and 21.0 h (14.8) in the VG and CS groups, respectively.

The sequencing successfully identified the microbial taxonomy of the gut in 89 (64.0%) samples of the VG and 44 (31.43%) samples of the CS. We used only these data for statistical analysis. The demographic data of the patients is presented in Tables 1(a) and 1(b). The mean (SD) maternal age was 27.9 (5.1) years old. There were significantly higher levels of income, education status, and underlying maternal disease in the CS. All mothers consumed all types of food except three cases in the CS with seafood allergies.

**Figure 4:**
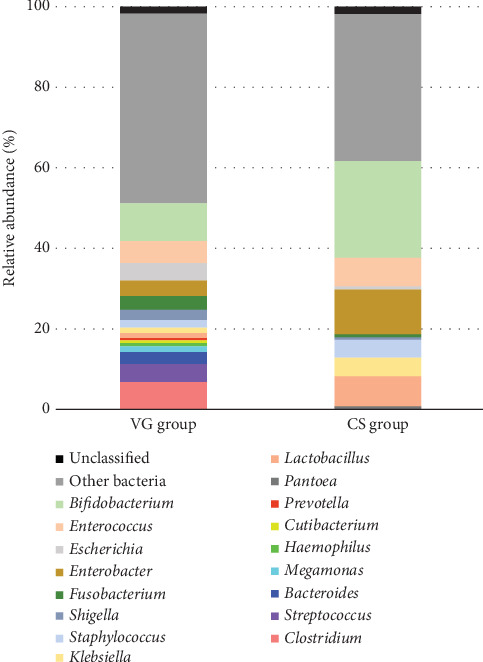
Genus of gut microbiome profiles generated by 16S rRNA full-length gene using Oxford nanopore sequencer. Eighty-nine samples of VG represented 16 bacteria genera with a percentage of relative abundance more than 0.5% and 44 samples of CS represented 10 bacteria genera with a percentage of relative abundance more than 0.5%.

The 16S rRNA sequencing results revealed higher total gut microbiome reads in the VG (7,019,452) than in the CS (3,359,444). Of those reads, 6,897,967 (98.27%) and 3,299,355 (98.21%) were classified as microbiomes in the VG and CS groups, respectively. The diversity of the neonates' gut microbiomes was greater in those born via vaginal delivery than those born via cesarean delivery. Of the species identified in this study, 17 (51.5%) were found exclusively in the VG, while nine (27.3%) were found exclusively in the CS. Only seven species (21.2%) were found in both groups (Figure 4).

**Figure 5:**
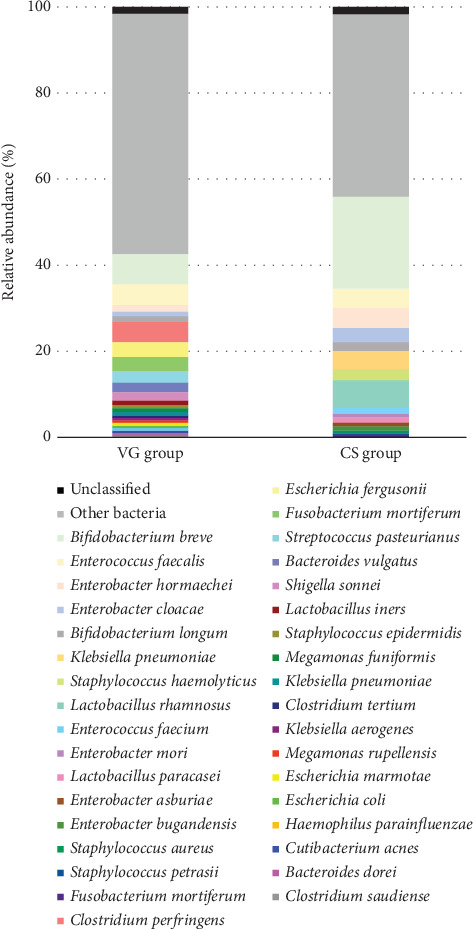
Species of gut microbiome profiles generated by 16S rRNA full-length gene using Oxford nanopore sequencer.

*Bifidobacterium* was the dominant genus in both the VG and CS groups. The proportion of *Bifidobacterium* was significantly higher in the CS (Table 2). Among all the *Bifidobacterium* species identified, *Bifidobacterium breve* was the most dominant, followed by *Bifidobacterium longum.* In the 89 samples of the VG, 24 bacteria species were found with a relative abundance percentage of more than 0.5%, and in the 44 samples of the CS, 16 bacteria species were found with a relative abundance percentage of more than 0.5%. (Figure 5). A significantly higher abundance of *Bifidobacterium* (*p* < 0.05) and *Enterobacter* (*p* < 0.05) species was found in the CS compared to the VG (Table 2).

In the VG, *Clostridium* (6.9%), *Enterococcus* (5.5%), *Escherichia* (4.5%), and *Streptococcus* (4.4%) were found to be the second most abundant genera in fecal specimens. Some, such as the genera *Fusobacterium*, *Streptococcus*, *Bacteroides*, *Megamonas*, and *Escherichia*, were found exclusively in the VG. In the CS, the most abundant genera after *Bifidobacterium* were *Enterobacter* (11.2%), *Lactobacillus* (7.8%), *Enterococcus* (7.2%), and *Klebsiella* (4.6%). However, opportunistic pathogens such as *Enterococcus*, *Klebsiella*, and *Clostridium* were found in both groups.

In the CS, there was no significant difference in neonatal gut microbiome abundance between those presenting with labor and those presenting without labor before cesarean delivery (Table 3).

## 4. Discussion

A lower diversity in gut microbiota was observed in neonates born via cesarean delivery compared to those born through vaginal delivery. *Bifidobacterium* spp. were predominant in both groups, with the abundance of a relatively higher proportion in the CS.

The diversity of gut microbiomes in neonates, which is significantly influenced by the mode of delivery, is consistent with findings from previous studies [10, 15]. A study conducted by Biasucci et al. in 2008, utilizing 16S rRNA methods and collecting fecal samples on the third day of life, indicated that the intestinal microbiota of neonates delivered by cesarean section appears less diverse regarding bacterial species. A later systematic review by Rutayisire et al. concluded that low total diversity of the gut microbiota during the first week of life was reported in infants delivered by cesarean section [16]. However, a recent study by Chu et al. in 2017 showed no difference in microbiota community function regardless of the delivery mode [17]. Studies on neonatal gut microbiomes exhibit variations such as timing of stool collection, sample size, and techniques used to identify bacterial genus/species. Furthermore, environmental and genetic factors also play a role in influencing individual gut microbiomes.

A significantly higher abundance of *Bifidobacterium* found in the CS is consistent with a study by Meghan et al. [7]. However, other studies have reported higher levels of *Bifidobacterium* in VGs [10, 18]. *Bifidobacterium* is an essential bacterium providing the most common genera in the infant gut microbiome. *B. breve* and *B. longum* are common species and are more prevalent during infancy than adulthood, especially during the first year of life. *Bifidobacterium* is involved in various physiological and immunological functions such as the digestion of human milk oligosaccharides, improving gut barrier function, and reducing intestinal permeability [19–21]. Lower levels of *B. longum* may be associated with allergic diseases such as atopic dermatitis [22, 23], but the outcome of such bacterial abundance has not been well stated. Some studies have shown the benefit of high levels of *Bifidobacterium*, while a decrease in *Bifidobacterium* colonization early in life may increase the risk of neonatal complications [24].

Consistent with previous studies, our study found lower levels of *Bacteroides* in the CS [7, 25], and several studies have shown delayed colonization of *Bacteroides* [23, 25, 26]. *Bacteroides* is the major genus found in adults, but these babies had lower *Bacteroides* levels since they lacked exposure to it during cesarean delivery. This may result in a negative impact on infant immune development, maintenance of intestinal homeostasis [27], and, later, food digestion [28].

In this study, *Staphylococcus aureus* (*S. aureus*) was higher in the CS, consistent with the study of Shao et al. [25]. The proposed source of *S. aureus* colonization was maternal skin flora [29]. Intestinal colonization with *S. aureus* is associated with higher levels of inflammation, which contribute to the development of inflammatory diseases such as asthma and allergies, including atopic dermatitis or atopic eczema [30], and food allergies [31]. We also identified higher levels of *Clostridium perfringens* in the VG, comprising 5.08% of all identified species, consistent with a previous study [7]. This may be attributed to initial gut colonization from maternal vaginal and fecal microbiota. Association with a higher risk of developing necrotizing enterocolitis, especially in preterm infants [32, 33], and prolonged diarrhea [34] has been found.

Our study had several strengths. Firstly, there were few studies reporting data on the differences in gut microbiome between neonates delivered via vaginal delivery and those delivered by cesarean delivery in Thailand. Secondly, we collected all fecal samples from healthy neonates within 48 h, and neonates whose mothers had used antibiotics were not included in the study, except when prophylactic antibiotics had been used for cesarean delivery. Thirdly, the adjusted DNA extraction protocol can be used as an alternative technique in subsequent studies to create the proper meconium-specific protocol for analyzing a neonate's gut microbiome.

One limitation of our study was the potential confounding effect of breastfeeding practices on a neonate's gut microbiome. Thus, collection of the first passing of meconium may represent an opportunity to access the neonatal gut microbiome directly after delivery [35]. Second, we have not stated the details of cesarean delivery, such as presenting with membrane rupture before cesarean delivery. These variations may result in different neonatal gut microbiomes.

There were many explanations for this lower diversity. Firstly, there was a lack of transmission of gut microbiome from mother to child through the vaginal route. Secondly, there was a lack of or delayed colonization with *Bacteroides* [8, 36], which can be persistent [25] or delayed by up to 1 year in some infants [8]. And finally, empirical antibiotics were used in cesarean deliveries [37].

The reasons behind elevated *Bifidobacterium* levels in the gut microbiomes of cesarean-delivered infants in some studies are not yet fully understood [7, 10]. Although most studies associate vaginal delivery with higher *Bifidobacterium* levels, some studies present different findings [8, 28]. Potential explanations include the use of antibiotic prophylaxis for cesarean delivery, which could lead to a decrease in overall gut microbial diversity or selectively eliminate certain bacterial species [7, 8]. For instance, *Bifidobacterium* species may be sensitive only to penicillin administration, not cephalosporins [8, 28]. Nonetheless, conflicting results in other studies were observed. For instance, Jakosson et al. [8] did not observe a significant difference in infant gut microbiome between mothers who had received prophylactic antibiotics and those who did not. Yassour et al. demonstrated a lower level of *Bifidobacterium* species in infants born via cesarean section and exposed to antibiotics, including cefazolin, compared to infants not exposed to antibiotics [37]. Our institutional protocol is to administer cefazolin before making a skin incision on the mother in the CS. Cephalosporin does not affect maternal microbiomes [38]; however, there has been limited research on the specific effects of cefazolin on *Bifidobacterium* levels in the infant gut microbiome. Therefore, we cannot draw a definite conclusion regarding the influence of empirical antibiotic use on the abundance of *Bifidobacterium* in our study.

Another hypothesis suggests that early breastfeeding may influence *Bifidobacterium* levels in infants, as breastfed infants are more likely to have higher levels due to the utilization of oligosaccharides as a food source [1]. Cesarean-delivered infants are more likely to experience delays in breastfeeding initiation, potentially affecting *Bifidobacterium* abundance [39, 40]. However, in our hospital, there is a policy of early breastfeeding for all mothers, making it uncertain whether this could confound our study. One of the challenges in obtaining information about breastfeeding practices was the reliance on subjective self-reporting by mothers, which could introduce informative bias on aspects such as timing, frequency, and the amount of breast milk.

Several studies reported that more diversity of gut microbiota during the first few months of life is conversely linked with the risk of some allergic disease later in an individual life [14, 41, 42]. Encouragement for cesarean section only in indicated circumstances is still a standard practice. Currently, the use of probiotics to alter the gut microbiome can reduce complications in premature newborns, such as necrotizing enterocolitis and late-onset sepsis [43]; however, there is no established evidence of the probiotic's benefit on the alteration of gut microbiome in the term neonate. It is also interesting to explore the impact of antibiotic prophylaxis on the gut microbiome of infants born through cesarean delivery and its long-term health effects. Moreover, considering that gut microbiota diversity tends to diminish after 6 months of life [16], efforts should be directed toward identifying factors that restore healthy gut microbiota.

Our study has other limitations, with 47 (34.6%) and 96 (68.6%) samples in the VG and CS groups being unsuccessfully sequenced. This may be attributed to sterility or very low bacterial abundance, particularly in the CS, where the tar-like meconium posed extraction difficulties due to its low biomass microbiome and high PCR inhibitor concentration [44]. Meconium analysis lacks standardized sequencing techniques for microbial assessment of the gut. We adjusted our extraction protocol to enhance DNA yield, yet uninterpreted samples suggest a potentially minimal bacterial presence. Future research should employ additional techniques to achieve clearer conclusions.

## 5. Conclusions

Neonates born via vaginal delivery exhibited higher diversity but lower dominant bacterial abundance in the gut microbiome compared to those born via cesarean delivery. The dominant genus in neonates' gut microbiomes was *Bifidobacterium*, with a statistically significant higher abundance observed in infants born via cesarean delivery. Much evidence suggests that gut microbiome can influence future health; this highlights the careful consideration of the route of delivery that may affect individual well-being in the future. Moreover, our findings are fundamental to future investigation on replenishing neonatal gut microbiome in neonates born by cesarean section.

## Figures and Tables

**(a):** 

**Characteristic**	**Values**		**p** ** value**
	**Vaginal delivery (** **N** = 89**)**	**Cesarean delivery (** **N** = 44**)**	
Age (years) (mean [SD])	27.3 (5.2)	29.0 (4.8)	0.06
Gestational age (weeks) (mean ± SD)	39.0 (1.0)	38.9 (0.9)	0.55
BMI (kg/m^2^) (number [%])			0.07
< 18.5	6 (6.7)	0 (0)	
18.5–24.9	50 (56.2)	26 (59.1)	
25.0–29.9	22 (24.7)	10 (22.7)	
≧ 30.0	11 (12.4)	7 (15.9)	
≧ 40.0	0 (0)	1 (2.7)	
Parity (number [%])			0.65
0	36 (40.4)	19 (43.2)	
≧ 1	53 (59.6)	25 (56.8)	
Miscarriage (number [%])			0.27
No	81 (91.0)	36 (81.8)	
Yes	8 (9.0)	8 (18.2)	
Underlying disease (number [%])	4 (4.5)	9 (20.5)	< 0.01
Chronic hypertension	2 (2.3)	1 (2.3)	0.74
Diabetes	2 (2.3)	0 (0)	0.73
Dyslipidemia	0 (0)	2 (4.5)	0.32
Autoimmune disease	0 (0)	2 (4.5)	0.32
Heart disease	0 (0)	1 (2.3)	0.15
Thyroid disease	0 (0)	2 (4.5)	0.04
Chronic hepatitis B infection	0 (0)	1 (2.3)	0.15
Medications			0.79
Only ANC medication	79 (88.7)	42 (95.4)	
No medication	6 (6.7)	1 (2.3)	
No data	3 (3.4)	0 (0)	
Ethnicity (number [%])			0.57
Thai	69 (77.5)	35 (79.5)	
Myanmar	9 (10.1)	5 (11.4)	
Cambodian	9 (10.1)	4 (9.1)	
Laos	2 (2.3)	0 (0)	
Education			< 0.001
None	17 (19.1)	8 (18.2)	
Elementary school	5 (5.6)	4 (9.1)	
High school	46 (51.7)	17 (38.6)	
University	21 (23.6)	15 (34.1)	
Income (mean [SD])	15281.8 (8992.6)	20,274 (11859.7)	0.01
No data (number [%])	1 (1.1)	5 (11.4)	
Smoking (number [%])			0.75
No smoking	76 (85.4)	37 (82.1)	
Active smoking	5 (5.5)	2 (4.5)	
Passive smoking	8 (9.0)	5 (11.4)	
Alcohol drinking (number [%])			0.48
No	88 (98.9)	44 (100.0)	
Yes	1 (1.1)	0 (0)	
Illicit drug use (number [%])			—
No	89 (100.0)	44 (100.0)	
Yes	0 (0)	0 (0)	
Food preference (number [%])			0.07
All types of foods	89 (100.0)	41 (93.2)	
Allergic to seafood	0	3 (6.8)	
ANC risk (number [%])			
GDM	8 (9.0)	8 (18.2)	0.13
Anemia	27 (30.3)	14 (31.8)	0.86
Teenage pregnancy	2 (2.3)	1 (2.3)	0.99
Poor weight gain	5 (5.6)	0 (0)	0.11
Excessive weight gain	7 (7.9)	5 (11.4)	0.51
Poor ANC	7 (7.9)	1 (2.3)	0.20
No ANC	1 (1.1)	0 (0)	0.48
Preeclampsia	1 (1.1)	0 (0)	0.48
Maternal leiomyoma	1 (1.1)	0 (0)	0.48
Fetal growth restriction	2 (2.3)	2 (4.5)	0.47
Fetal large for gestation	0 (0)	2 (4.5)	0.04
Oligohydramnios	1 (1.1)	0 (0)	0.48

*Note:* Data are presented as mean ± standard deviation (SD) or number (%).

Abbreviation: BMI, body mass index.

**(b):** 

**Characteristic**	**Values**		**p** ** value**
	**Vaginal delivery (** **N** = 89**)**	**Cesarean delivery (** **N** = 44**)**	
Sex (number [%])			0.85
Male	44 (49.4)	21 (47.7)	
Female	45 (50.6)	23 (52.3)	
Birth body weight (g) (mean (SD))	3112.3 (332.8)	3214.0 (364.4)	0.09
APGAR score at 5 min (number [%])			—
7–10	89 (100.0)	44 (100.0)	
4–6	0 (0)	0 (0)	
0–3	0 (0)	0 (0)	
Amniotic fluid (number [%])			0.26
Clear	84 (94.4)	40 (90.9)	
Thin meconium	3 (3.3)	0 (0)	
Thick meconium	2 (2.3)	4 (9.1)	

*Note:* Data are presented as mean ± standard deviation (SD) or number (%).

Abbreviation: BMI, body mass index.

**Table 2:** Comparison of the relative abundance of taxon species between neonate's gut microbiome delivered via vaginal delivery and cesarean delivery.

**Taxon species level**	**VG (** **n** = 89**)**	**CS (** **n** = 44**)**	**p** ** value** ^a^
Bifidobacteriaceae (mean [SD])			
*Bifidobacterium breve*	7.0 (1.9)	26.0 (5.2)	< 0.05
*Bifidobacterium longum*	0.9 (0.2)	2.6 (0.5)	< 0.05
Enterobacteriaceae (mean [SD])			
*Enterobacter cloacae*	0.7 (0.1)	2.7 (0.7)	< 0.05
*Enterobacter hormaechei*	1.0 (0.4)	3.7 (1.0)	< 0.05
Enterococcaceae (mean [SD])			
*Enterococcus faecalis*	2.3 (1.2)	4.1 (2.2)	0.439

^a^Determined by the independent *T*-test with unequal variances.

**Table 3:** Comparison of the relative abundance of taxon species between neonates' gut microbiome delivered via cesarean delivery with and without labor.

**Taxon species level**	**With labor (** **n** = 26**)**	**Without labor (** **n** = 18**)**	**p** ** value**
Bifidobacteriaceae (mean [SD])			
*Bifidobacterium breve*	28.5 ± 7.2	24.0 ± 8.0	0.681
*Bifidobacterium longum*	2.8 ± 3.4	2.3 ± 3.1	0.334
Lactobacillaceae (mean [SD])			
*Lactobacillus rhamnosus*	3.5 ± 2.7	11.2 ± 5.6	0.191
Enterobacteriaceae (mean [SD])			
*Enterobacter hormaechei*	2.3 ± 0.9	5.9 ± 1.9	0.063
*Enterobacter cloacae*	1.8 ± 0.7	4.0 ± 1.3	0.124
*Enterobacter mori*	0.9 ± 0.4	0.6 ± 0.2	0.510
*Enterobacter asburiae*	0.7 ± 0.2	1.0 ± 0.3	0.403
*Enterobacter bugandensis*	0.5 ± 0.2	0.9 ± 0.3	0.253
Enterococcaceae (mean [SD])			
*Enterococcus faecalis*	6.4 ± 3.9	0.5 ± 0.4	0.204
*Enterococcus faecium*	1.4 ± 1.4	1.9 ± 1.7	0.822

## Data Availability

The data that support the findings of this study are available on request from the corresponding author (Wipada Laosooksathit) upon reasonable request.

## Supporting Information

Supporting Information Additional supporting information can be found online in the Supporting Information section. We already included genomic DNA (gDNA) extraction by ZymoBIOMICS DNA Miniprep Kit protocol in the supporting information.
