# Supplementary material for: Comparison of Gut Microbiomes Between Neonates Born by Cesarean Section and Vaginal Delivery: Prospective Observational Study
Source: Biomed Res Int. 2024 Nov 28;2024:8302361. doi: 10.1155/bmri/8302361 (PMC11620805; doi:10.1155/bmri/8302361)
Supplement: Supporting Information — Additional supporting information can be found online in the Supporting Information section. We already included genomic DNA (gDNA) extraction by ZymoBIOMICS DNA Miniprep Kit protocol in the supporting information. [file 8302361.f1.docx]

**Supplementation**

**Genomic DNA (gDNA) extraction by ZymoBIOMICS DNA Miniprep Kit protocol**

1. Add 1 ml of each sample to ZR BashingBead^TM^ Lysis Tubes.
2. Secure the bead beater fitted with a 2 ml tube holder and process using optimized beat beating condition. Then centrifuge the Bead lysis tube at 10,000 x g for 1 minute.
3. Transfer 400 ul of the supernatant to the Zymo-SpinTM III-F Filter in a collection tube and centrifuge at 8,000 x g for 1 minute and discard the filter.
4. Add 1,200 ul of ZymoBIOMICSTM DNA Binding Buffer to the filtrate in the collection tube and mix well.
5. Transfer 800 ul of the mixture into a Zymo-SpinTM IICR Column in a collection tube and centrifuge at 10,000 x g for 1 minute, then discard the flow-through.
6. Transfer the remaining 800 ul of the sample into the same column and centrifuge at 10,000 x g for 1 minute, then discard the flow-through.
7. Add wash buffer to the Zymo-SpinTM IICR Column then centrifuge at 10,000 x g for 1 minute for each buffer subsequently (5.1-5.3):
   1. ZymoBIOMICSTM DNA Wash Buffer 1 - 400 ul
   2. ZymoBIOMICSTM DNA Wash Buffer 2 - 700 ul
   3. ZymoBIOMICSTM DNA Wash Buffer 2 - another 200 ul
8. Discard the flow-through after centrifugation for each buffer.
9. Transfer the Zymo-SpinTM IICR Column to a new microcentrifuge tube and add 50 ul of ZymoBIOMICSTM DNase/RNase-free water directly to the column matrix and incubate for 1 minute. Then centrifuge at 10,000 x g for 1 minute to elute the DNA.
10. Prepare the Zymo-SPINTM III-HRC by adding 600 ul of ZymoBIOMICSTM HRC Prep Solution and centrifuge at 8,000 x g for 3 minutes.
11. Transfer the eluted DNA to the prepared Zymo-SPINTM III-HRC filter in a clean 1.5 ml microcentrifuge tube and centrifuge at exactly 16,000 x g for 3 minutes.

Adjusted step

1. Transfer the Zymo-Spin^TM^ IICR Column to a dry collection tube and centrifuge at 10,000 x g for 5 minutes.
2. Transfer the Zymo-Spin^TM^ IICR Column to a new microcentrifuge tube and add 50 ul of ZymoBIOMICS^TM^ DNase/RNase-free water directly to the column and incubate for 5 minutes. Then centrifuge at 10,000 x g for 1 minute to elute the DNA.
3. Transfer the sample into new microcentrifuge.
